# Benmelstobart+anlotinib: an emerging therapeutic option in the targeted-immunotherapy era

**DOI:** 10.3389/fonc.2026.1748214

**Published:** 2026-04-13

**Authors:** Pan Cheng, Hong Gu, Qingqing Chen, Guangyu Zhao, Jichen He

**Affiliations:** 1 Department of Pharmacy, Jingjiang People’s Hospital Affiliated to Yangzhou University, Taizhou, China; 2 Department of Pharmacy, Jiangsu Province Hospital, Nanjing, China

**Keywords:** anlotinib, benmelstobart, immune checkpoint inhibitor, malignant tumors, programmed cell death ligand 1

## Abstract

In recent years, with the gradual deepening of research, basic and clinical studies on targeted-immunotherapy combination regimens have flourished and yielded exciting results. The novel programmed cell death ligand 1 (PD-L1) inhibitor benmelstobart in combination with the targeted agent anlotinib demonstrates synergistic antitumor effects, achieving a synergy therapeutic outcome. This article focuses on the mechanisms underlying the combined action of “benmelstobart+anlotinib”, summarizes the currently approved indications and clinical research progress, and aims to provide alternative therapeutic options for patients with malignant tumors as well as new insights for the development and selection of future combination treatment strategies.

## 1 Introduction

Benmelstobart (TQB2450) is a novel humanized immunoglobulin (Ig) G1 subtype anti-programmed death-ligand 1 (PD-L1) antibody independently developed in China. Benmelstobart binds specifically to PD-L1, blocking its binding to programmed death receptor 1 (PD-1), thereby activating T cell activity and producing a sustained anti-tumor effect (1). Following demonstration of its survival benefits and safety in clinical trials, benmelstobart received approval from China’s National Medical Products Administration (NMPA) on April 30, 2024, and has subsequently been approved for small cell lung cancer, endometrial cancer, and kidney cancer as of June 1, 2025 (2–4). Currently, clinical trials have also been initiated for benmelstobart in various malignant tumors, including non-small cell lung cancer (NSCLC), soft tissue sarcoma, breast cancer, ovarian cancer, and tumors of the digestive system. Research has shown that the anti-angiogenic drug anlotinib can synergize with benmelstobart to enhance anti-tumor effects. Consequently, most current clinical studies on benmelstobart are focused on combination therapy regimens involving “benmelstobart + anlotinib”. This article reviews the research progress of benmelstobart in the treatment of malignant tumors.

## 2 Pharmacological mechanism study of “benmelstobart + anlotinib”

Immune checkpoint inhibitors (ICIs) are a class of inhibitory molecules that modulate immune response strength. The PD-1/PD-L1 pathway is a key immune checkpoint mechanism, frequently exploited by tumor cells to evade immune surveillance. PD-L1 inhibitors offer an advantage over PD-1 inhibitors by avoiding blockade of the PD-L2 pathway, thereby preserving immune regulatory functions, while also effectively blocking both PD-1 and B7.1 to enhance T-cell activation (5). Natural IgG1 is the most commonly used therapeutic antibody because it has higher affinity and stability than other IgG subtypes. However, its strong Fc segment effector function may lead to the killing of immune cells in the tumor microenvironment, necessitating engineering modifications to reduce Fc-mediated effects (6). Compared to other PD-L1 inhibitors, benmelstobart is modified at only a single specific site, preserving the natural structural advantages of IgG1’s high stability. The molecular design of benmelstobart incorporates specific amino acid mutations in the Fc region to eliminate antibody-dependent cell-mediated cytotoxicity (ADCC) and complement-dependent cytotoxicity (CDC) effects, thereby reducing immune-related cell depletion. This Fc modification prevents the Fc effector functions from causing unintended damage to or misdirected killing of immune cells. It also enhances immune activation and maximizes the antitumor activity of benmelstobart (7).

**Figure 1:**
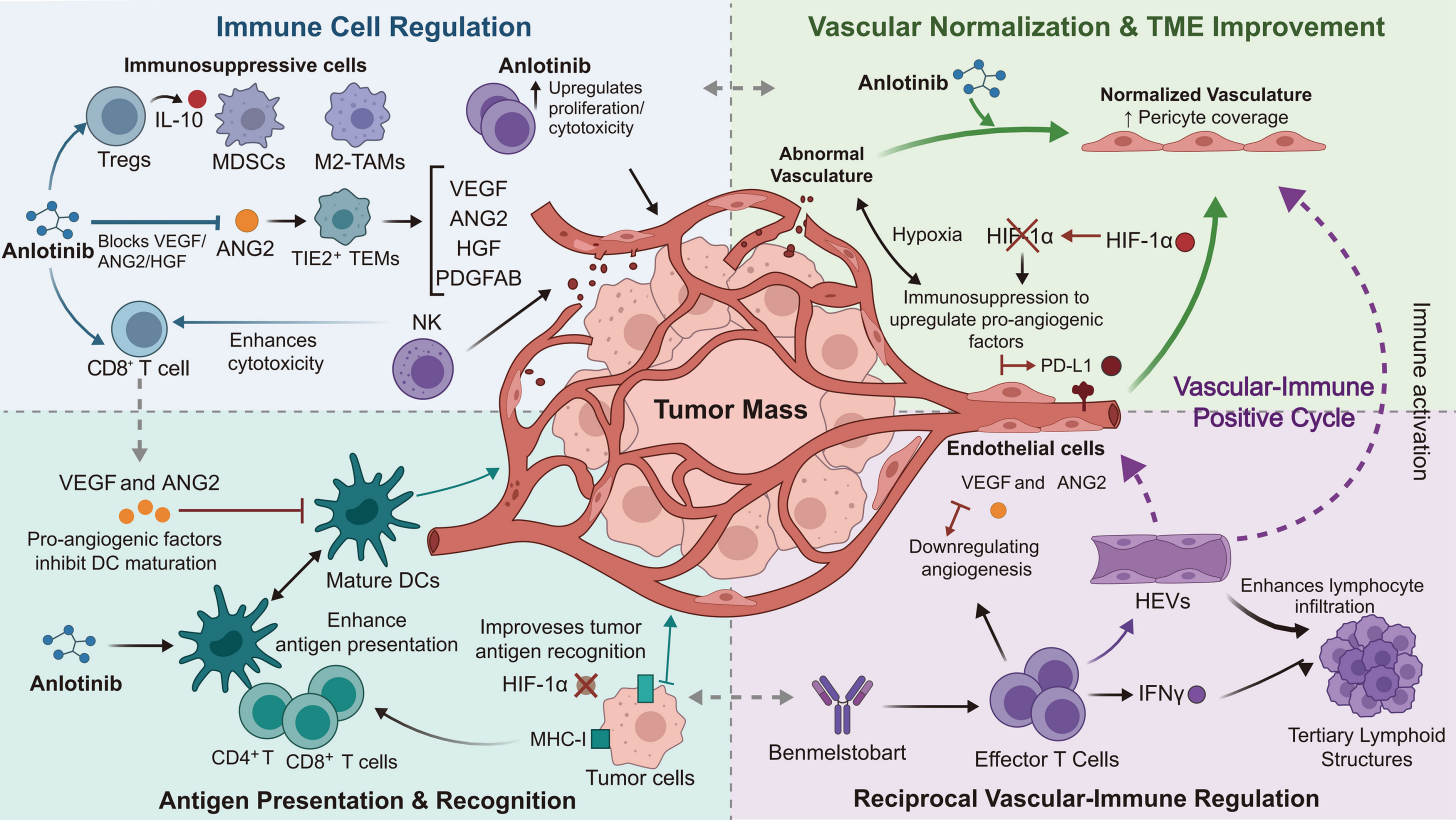
Schematic illustration of the synergistic antitumor mechanisms benmelstobart combined with anlotinib. Immune Cell Regulation: Anlotinib inhibits the recruitment and proliferation of immunosuppressive cells (Tregs, MDSCs, and M2-TAMs). Simultaneously, it enhances the proliferation and cytotoxicity of effector CD8+ T and NK cells by reversing VEGFR-mediated inhibition. Vascular Normalization: Anlotinib facilitates the transition to normalized vasculature with increased pericyte coverage. This normalization alleviates hypoxia through inhibition and downregulates PD-L1 expression on tumor and endothelial cells, further reducing immunosuppression. Reciprocal Regulation: Benmelstobart activates effector T cells to secrete, which induces vascular remodeling and the formation of high endothelial venules (HEVs) and tertiary lymphoid structures. This establishes a “Vascular-Immun Positive Cycle” that sustains lymphocyte infiltration. Antigen Presentation: Anlotinib restores DC maturation by overcoming pro-angiogenic factor inhibition. Furthermore, vascular normalization and inhibition upregulate MHC-I expression on tumor cells, significantly improving tumor antigen presentation and recognition by T cells. ANG2, Angiopoietin 2; DCs, Dendritic cells; HEVs, High endothelial venules; HGF, Hepatocyte growth factor; HIF-1α, Hypoxia-inducible factor 1-alpha; IFNγ, Interferon gamma; M2-TAMs, M2-type tumor-associated macrophages; MDSCs, Myeloid-derived suppressor cells; MHC-I, Major histocompatibility complex class I; NK cells, Natural killer cells; PD-L1, Programmed death-ligand 1; PDGFAB, Platelet-derived growth factor AB; TME, Tumor microenvironment; Tregs, Regulatory T cells; VEGF, Vascular endothelial growth factor; VEGFR, Vascular endothelial growth factor receptor.

Anlotinib is a multi-target tyrosine kinase inhibitor (TKI) with anti-angiogenic activity, primarily mediated through the inhibition of VEGFR and PDGFR pathways (8). It suppresses tumor neovascularization by blocking VEGF signaling and promotes vascular normalization, which improves drug delivery and immune cell infiltration (9, 10). Additionally, anlotinib modulates the extracellular matrix via the RhoA/ROCK pathway to enhance intratumoral drug distribution (11). Research indicates that abnormal tumor vasculature creates an immunosuppressive microenvironment, while anti-angiogenic therapy can normalize vessels and alleviate immunosuppression, thereby enhancing immune cell activity. Conversely, immunotherapy may augment antigen release and immune activation, while also reducing VEGF production to further support the anti-angiogenic effect of anlotinib (12). Thus, the combination of anlotinib and benmelstobart produces a synergistic “immune activation + vascular suppression” effect, offering a synergy therapeutic outcome (**Figure 1**) (13, 14).

## 3 Clinical efficacy of “benmelstobart + anlotinib” in different types of malignant tumors

### 3.1 Endometrial cancer

The incidence of endometrial cancer continues to rise, making it a significant threat to women’s health. In recent years, the rapid advancement of precision medicine, including immunotherapy and targeted therapy, has provided new treatment options for patients with endometrial cancer (15). Due to the significant clinical benefit demonstrated by pembrolizumab plus lenvatinib in patients with advanced/recurrent non-deficient mismatch repair (dMMR) endometrial carcinoma who had received at least two prior lines of therapy, the U.S. FDA granted accelerated approval for this combination in endometrial cancer. This indication, however, has not yet been approved in China. Moreover, in this study, grade 3 or higher treatment-related adverse events (TRAEs) occurred in 90.1% of patients receiving pembrolizumab plus lenvatinib, with treatment discontinuations due to adverse events reaching 31.5%, highlighting a toxicity profile that requires careful consideration (16).

To explore effective later-line therapies for endometrial cancer, a phase II clinical trial (TQB2450-II-08) led by Professor Xiaohua Wu from Fudan University Shanghai Cancer Center is evaluating benmelstobart combined with anlotinib in patients with recurrent or metastatic disease (3). This multicenter, open-label, multi-cohort phase II study included patients stratified into three cohorts: Cohort 1 (non-MSI-H/dMMR; benmelstobart plus anlotinib), Cohort 2 (MSI-H/dMMR; benmelstobart monotherapy), and Cohort 3 (non-MSI-H/dMMR; anlotinib monotherapy). Data presented at the 2024 ASCO Annual Meeting (cutoff date: November 9, 2023) demonstrated an objective response rate (ORR) of 31.76% in Cohort 1, 34.38% in Cohort 2, and 22.58% in Cohort 3. The median progression-free survival (mPFS) was 8.38 months, 8.21 months, and 6.80 months, and the median overall survival (mOS) was 21.72 months, 27.66 months, and 16.03 months for Cohorts 1, 2, and 3, respectively. Updated data presented at the 2024 International Gynecologic Cancer Society (IGCS) Annual Global Meeting (cutoff date: May 9, 2024) showed that in Cohort 1, the ORR was 34.12%, the mPFS was 8.80 months, and the mOS was 21.78 months. The combination of benmelstobart and anlotinib demonstrated promising survival outcomes in patients with non-MSI-H/non-dMMR tumors (17).

Furthermore, a recent randomized, open-label phase II clinical trial evaluated the efficacy of benmelstobart plus carboplatin/paclitaxel with or without anlotinib, followed by benmelstobart with or without anlotinib maintenance therapy, as first-line treatment for patients with advanced or recurrent endometrial cancer. As of November 1, 2024, 71 patients had been enrolled: 38 in the benmelstobart + anlotinib + chemotherapy group and 33 in the benmelstobart + chemotherapy group. The median follow-up times were 16.2 months and 14.2 months, respectively. The benmelstobart + anlotinib + chemotherapy group achieved an ORR of 86.1% (95% CI: 70.5–95.3), compared with 80.6% (95% CI: 62.5–92.5) in the benmelstobart + chemotherapy group. Compared to the benmelstobart + chemotherapy group, the benmelstobart + anlotinib + chemotherapy group demonstrated a significant PFS benefit (hazard ratio [HR] 0.38, 95% CI: 0.18–0.81; mPFS: not reached vs. 8.41 months). Median OS was not reached in either group (HR = 0.29, 95% CI: 0.07–1.16). A PFS benefit was also observed in the pMMR subgroup (HR = 0.35, 95% CI: 0.15–0.79) (18). The regimen demonstrated clinically meaningful improvements in ORR and PFS in previously untreated patients with advanced or recurrent endometrial cancer. This treatment strategy showed particularly pronounced benefits in improving outcomes for patients with pMMR tumors, representing a potential new treatment option for this population.

### 3.2 Renal cell carcinoma

The treatment of RCC has progressed from single-agent targeted therapy into the era of immunotherapy combined with targeted agents. Regimens such as dual immunotherapy combinations and immune-targeted doublets are gradually becoming the standard of care (19, 20). For example, the RENOTORCH study demonstrated a significant efficacy advantage of toripalimab combined with axitinib over sunitinib monotherapy (21). This treatment strategy has now been approved by the NMPA for first-line treatment of advanced RCC. However, due to factors such as drug accessibility, cost, and safety concerns, some patients with RCC still do not receive effective treatment, highlighting the need for providing more therapeutic options.

The ETER100 study (NCT04523272) is a randomized, open-label, active-controlled, multicenter phase III clinical trial. The trial enrolled 531 treatment-naïve patients with locally advanced or metastatic clear cell RCC, who were randomly assigned to two groups: the combination therapy group receiving benmelstobart plus anlotinib (*n* = 264) and the sunitinib monotherapy group (*n* = 267). The study demonstrated that compared with the sunitinib group, the benmelstobart plus anlotinib combination group achieved significantly longer mPFS (18.96 *vs.* 9.76 months; HR = 0.53, *P* < 0.0001) and a higher ORR (71.6% *vs.* 25.1%; *P* < 0.0001), while the mOS in the overall population had not yet been reached in either group (4). Based on current results, the benmelstobart plus anlotinib combination demonstrated a near doubling of mPFS survival compared with the control group, representing a statistically significant and clinically meaningful improvement. The ETER-100 study demonstrated the most favorable efficacy data reported to date among trials evaluating PD-L1 inhibitor-based combinations and showed comparable efficacy to previous PD-1 inhibitor-based combination regimens, providing robust evidence to support the use of frontline PD-L1 inhibitor plus targeted therapy combinations in advanced RCC.

### 3.3 Extensive stage small cell lung cancer

SCLC is generally characterized by short doubling time, high growth fraction, marked heterogeneity, and early invasive propensity. Although the success of the IMpower133 and CASPIAN trials has established immune-checkpoint inhibitors combined with chemotherapy as the standard first-line treatment for ES-SCLC, the ORR, PFS, and OS remain suboptimal (22, 23). Results from the phase III ETER701 trial demonstrate that the combination anlotinib plus benmelstobart and chemotherapy has promising efficacy in this setting (24). The ETER701 study was a multicenter, randomized, double-blind, parallel-controlled, phase III trial (NCT04234607) conducted from March 2020 to December 2021 across 72 centers in China. The study enrolled 738 patients to evaluate the efficacy and safety of benmelstobart plus anlotinib and etoposide-carboplatin (EC) versus placebo plus anlotinib-EC versus placebo-EC as first-line treatment for ES-SCLC. The co-primary endpoints were PFS and OS. Secondary endpoints included investigator-assessed PFS, ORR, disease control rate (DCR), duration of response (DOR), and safety. At a median follow-up duration of 14 months, median PFS was significantly longer with the quadruplet regimen (6.9 months versus 4.2 months with chemotherapy; HR = 0.32, 95% CI: 0.26-0.41; *P* < 0.0001) and the triplet regimen (5.6 months versus 4.2 months; HR = 0.44, 95% CI: 0.36-0.55; *P* < 0.0001). At the prespecified interim analysis for OS, median durations were significantly longer with the quadruplet regimen (19.3 months versus 11.9 months; HR = 0.61, 95% CI: 0.47-0.79; *P* = 0.0002) but not with the triplet regimen (13.3 months; HR = 0.86, 95% CI: 0.67-1.10; *P* = 0.17) (2). This regimen represents the first successful application of a novel therapeutic approach combining immunotherapy, multi-target anti-angiogenic agents, and chemotherapy in ES-SCLC, providing a new treatment strategy for patients. Currently, it has been endorsed by both the Chinese Society of Clinical Oncology (CSCO) Guidelines for Small Cell Lung Cancer (2025 edition) and the Expert Consensus on Immunotherapy for Small Cell Lung Cancer (2025 edition) as a preferred first-line treatment option for ES-SCLC (25, 26). We anticipate its future incorporation into international guidelines, potentially establishing a new global standard of care for SCLC treatment.

A network meta-analysis of 10 randomized controlled trials (involving 5544 patients; drug combinations included adebrelimab, atezolizumab, durvalumab, durvalumab plus tremelimumab, ipilimumab, pembrolizumab, serplulimab, benmelstobart plus anlotinib, tislelizumab, tiragolumab plus atezolizumab, and toripalimab in combination with chemotherapy) was conducted. The analysis revealed that the regimen containing benmelstobart, anlotinib, and chemotherapy had the highest probability of achieving the best PFS and OS, compared to chemotherapy alone. Compared with ICI plus chemotherapy, this regimen also achieved significantly better PFS and showed a trend toward improved OS (27). Yan et al. explored the cost-effectiveness of benmelstobart combined with anlotinib and chemotherapy (B+A+EC) as a first-line treatment for ES-SCLC from the perspective of China’s healthcare system (28). The results demonstrated that neither the B+A+EC nor the A+EC regimen was cost-effective at current drug prices in China. Price reductions for benmelstobart and anlotinib may improve the cost-effectiveness of these therapeutic regimens.

### 3.4 Limited-stage small-cell lung cancer

Approximately 30% of patients with SCLC present with LS-SCLC. Treatment advances in LS-SCLC have been relatively slow, with management primarily relying on conventional chemotherapy and radiotherapy. Yu et al. conducted a prospective, single-arm, phase Ib study aimed to evaluate the efficacy and safety of benmelstobart combined with anlotinib as maintenance therapy for LS-SCLC following concurrent or sequential chemoradiotherapy (CCRT or SCRT) (29). Patients who did not show disease progression after chemoradiotherapy were enrolled. They received benmelstobart and anlotinib every 3 weeks for up to 24 months. Benmelstobart was intravenously administered at a dose of 1200mg every 3 weeks. Anlotinib was initiated at a dose of 8 mg daily for days 1-14; if well tolerated, the dose was increased to 10mg. Fifteen patients were enrolled in the study between May 31, 2023 and October 13, 2023. As of October 31, 2024, the median follow-up time was 15.13 months. The 12-month PFS rate was 86.7% (95% CI: 71.1-100.0), and the OS rate at 12 months was 100%. The disease control rate was 100%. AEs were reported in 13 patients (86.67%), with fatigue being the most common treatment related AE (40.00%). And two SAEs were observed (elevation in cardiac troponin T and cerebral infarction), which were determined to be unlikely unrelated to the trial drugs. Radiation pneumonitis (RP) occurred in three patients, all classified as grade 2, and one patient developed grade 1 immunerelated pneumonitis. No grade 5 AEs occurred, and no patients withdrew from the study due to AEs. The findings of this study indicate that benmelstobart plus anlotinib holds promise as a maintenance therapy for patients with LS-SCLC.

### 3.5 Non-small cell lung cancer

#### 3.5.1 Advanced squamous non-small cell lung cancer

Currently, platinum-based doublet chemotherapy combined with ICIs serves as the backbone regimen for the systemic treatment of advanced sq-NSCLC (30). However, clinical evidence indicates that the cumulative toxicity induced by this immunochemotherapy combination (e.g., grade III-IV hematologic toxicity, neurotoxicity, and organ dysfunction) significantly limits subsequent treatment tolerance. A multicenter, randomized, double-blind, parallel-group phase III clinical trial (TQB2450-III-12) designed to evaluate the efficacy and safety of benmelstobart combined with chemotherapy followed by sequential combination with anlotinib versus tislelizumab combined with chemotherapy as first-line treatment for advanced sq-NSCLC. The study enrolled 579 patients with stage IIIB to IV sq-NSCLC who had not received prior systemic therapy and had no fatal bleeding risk before treatment. Patients were randomly randomized to the following two groups: Experimental group: Induction therapy with benmelstobart plus carboplatin and paclitaxel, followed by maintenance therapy with benmelstobart plus anlotinib; Control group: Induction therapy with tislelizumab plus carboplatin and paclitaxel, followed by maintenance therapy with tislelizumab plus placebo. Post-treatment efficacy data revealed that mPFS was 10.12 months in the experimental group and 7.79 months in the control group, representing a statistically significant improvement in the experimental arm. Notably, the PFS benefit was more pronounced in patients with PD-L1, TPS 1-49% (10.12 months *vs.* 7.00 months). In patients with TPS≥50%, mPFS was not reached in either group. The ORR was 71.94% in the experimental group and 65.14% in the control group. mDoR was longer in the experimental group (9.69 months; 95% CI: 8.44-not estimable [NE]) compared to the control group (8.34 months; 95% CI: 5.78-NE), with a hazard ratio of 0.58 (95% CI: 0.38-0.88; *P* = 0.0091). In terms of safety, 61.57% and 51.06% of patients in the experimental group and the control group, respectively, experienced grade≥3 adverse events related to benmelstobart/tislelizumab or anlotinib/placebo. There was no difference in the incidence of grade 5 TRAEs between the two groups (5.69% in the experimental group *vs.* 5.63% in the control group). The proportions of patients who discontinued any study drug due to TRAEs were 4.27% in the experimental group and 5.28% in the control group, respectively (31).

#### 3.5.2 Advanced mutated NSCLC

In Asian patients with advanced NSCLC, the EGFR mutation rate is as high as 51.4% (32). Platinum-based doublet chemotherapy with or without bevacizumab has become the standard-of-care for EGFR TKI-resistant, EGFR mutation-positive advanced NSCLC patients. However, given the significant toxicity and suboptimal treatment adherence associated with chemotherapy, there remains an unmet need for safer, more convenient chemotherapy-free regimens with comparable efficacy in this patient population following EGFR TKI resistance (33). The ALTER-L038 study is a multicenter, single-arm, open-label phase I/II trial designed to evaluate the efficacy and safety of benmelstobart plus anlotinib in patients with NSCLC following EGFR-TKI resistance. The study comprised two parts: The phase I portion employed a standard “3 + 3” dose-escalation design. Cohorts received benmelstobart (1200 mg) combined with escalating doses of anlotinib (8mg, 10mg, 12mg) to determine the maximum tolerated dose of anlotinib. The phase II portion enrolled 55 patients with advanced EGFR mutation-positive NSCLC who had experienced treatment failure with EGFR-TKIs. These patients received the combination of benmelstobart and anlotinib, with the confirmed recommended phase II dose (RP2D) established as anlotinib (12mg) plus benmelstobart (1200 mg). The study results demonstrated that the primary endpoint in Phase II, mPFS reached 9.0 months. Regarding secondary endpoints, mOS exceeded 28.9 months. Additionally, the ORR was 25.5% (95% CI: 14.7%-39.0%), the DCR was 87.3% (95% CI: 75.5%-94.7%), and the median duration of response was 19.8 months (95% CI: 7.7-26.2 months). Regarding safety, the incidence of grade 3–4 TRAEs was 25.5%. The most frequent TRAEs included hypertension (45.5%), palmar-plantar erythrodysesthesia syndrome (38.2%), and proteinuria (27.3%). irAEs occurred in 69.1% of patients, with only 10.9% being grade≥3 events (34).

Another randomized, single-blind, multicenter phase III clinical trial (CAMPASS) was designed to evaluate the efficacy and safety of benmelstobart plus anlotinib versus pembrolizumab as first-line therapy in patients with PD-L1-positive advanced NSCLC. The study enrolled patients with locally advanced or recurrent/metastatic NSCLC who were PD-L1 expression-positive (Tumor Proportion Score [TPS]≥1%) and had not received prior systemic therapy. Participants were randomized in a 2:1 ratio to receive either benmelstobart plus anlotinib or pembrolizumab plus placebo. The primary endpoint was PFS. Secondary endpoints included ORR, OS and DCR. As of the data cutoff date (May 20, 2023), the median follow-up for PFS was 11.4 months in the benmelstobart plus anlotinib (benmel+anlo) group and 10.6 months in the pembrolizumab plus placebo (pem+placebo) group. The study met its primary endpoint that the median PFS was significantly prolonged to 11.0 months (95% CI 9.2-12.6) in the benmel+anlo arm compared with 7.1 months (95% CI 5.8-9.5) in the pem+placebo arm (*P* = 0.007). Regarding secondary endpoints, the ORR was also significantly higher in the benmel+anlo group (57.3% *vs*. 39.6%; *P* = 0.001). Furthermore, the DCR were 85.9% in the benmel+anlo group and 79.1% in the pem+placebo group (*P* = 0.047). In terms of safety, the combination group of benmel+anlo exhibited manageable safety profiles and good tolerability. In the benmel+anlo group and the pem+placebo group, 98.3% and 88.1% of patients, respectively, experienced at least one TRAE. Among these, the incidence rates of grade 3 TRAEs in the two groups were 58.5% and 29.0%, respectively. Regarding treatment discontinuation, only 5.7%/3.7% of patients permanently discontinued benmelstobart/anlotinib, whereas the proportions of patients who discontinued pembrolizumab/placebo due to TRAEs were 8.0%/2.3% (35).

#### 3.5.3 Locally advanced unresectable NSCLC

The R-ALPS study is a randomized, double-blind, placebo-controlled, multicenter phase III clinical trial evaluating benmelstobart with or without anlotinib as maintenance therapy in patients with locally advanced unresectable (stage III) NSCLC who did not progress after concurrent or sequential chemoradiotherapy. This study enrolled 553 patients with locally advanced, unresectable stage III NSCLC (without disease progression after concurrent/sequential chemoradiotherapy [60Gy ± 10%]) and randomly assigned them to three groups: 1) benmelstobart (1200 mg, intravenous infusion, every 3 weeks) plus anlotinib (8 mg, oral, days 1-14, every 3 weeks); 2) benmelstobart monotherapy; 3) placebo group. Sample size allocation: 1:1:1 in the first stage and 1:1 in the second stage. As of the data cutoff date (November 30, 2023), the mPFS for the primary endpoint was 15.1 months (95% CI: 9.4-21.7) in the combination therapy group, which was significantly longer than the 4.2 months in the placebo group (HR = 0.49, 95%CI: 0.36-0.66, *P* < 0.0001). The mPFS in the monotherapy group was 9.7 months (95%CI: 6.0–34.4), which was also significantly longer than that in the placebo group (HR = 0.53, 95%CI: 0.39-0.72; Log-rank *P* < 0.0001). The 12-month PFS rate was 54.9% in the combination therapy group, 45.7% in the monotherapy group, and 26.4% in the placebo group. As of the data cutoff date (July 8, 2024), the mPFS was 17.4 months (95%CI: 12.5-24.8) in the combination therapy group and 11.2 months (95%CI: 7.0-20.7) in the monotherapy group (HR = 0.82, 95%CI: 0.63-1.08; Log-rank *P* = 0.1218) (36). The combination of benmelstobart and anlotinib leverages synergistic “immuno-antiangiogenic” mechanisms to provide an effective, chemotherapy-sparing therapeutic option for patients with PD-L1-high NSCLC and squamous histology, particularly those seeking chemotherapy-free regimens.

### 3.6 Triple negative breast cancer

Chemotherapy and immunotherapy constitute the primary first-line treatment approaches for TNBC. However, these modalities confer clinical benefits to only a minority of patients with advanced disease, highlighting an urgent unmet clinical need for more effective therapeutic strategies (37). Wang et al. investigated the safety and preliminary efficacy of benmelstobart plus anlotinib in patients with advanced TNBC (NCT03855358). Patients with advanced TNBC who received at least one line of systemic therapy with anthracyclines and/or taxanes were enrolled in the dose-escalation and dose-expansion cohorts. Between May 29, 2019, and September 28, 2020, 34 patients were enrolled (three in the dose-escalation cohort and 31 in the dose-expansion cohort). The ORR was 26.5% (95% CI: 12.9-44.4), and the DCR was 73.5% (95% CI: 55.6-87.1). Median PFS was 5.6 months (95% CI: 2.9-7.5), while mOS was not reached. TRAEs of grade ≥3 occurred in 17 patients (50.0%). The most common grade ≥3 TRAEs were QT interval prolongation (17.6%) and hypertension (14.7%). No treatment-related deaths were reported. Benmelstobart combined with anlotinib, as a chemotherapy-free regimen, demonstrated promising efficacy and manageable safety in previously treated patients with advanced TNBC (38). Additionally, within this study, 31 patients underwent targeted next-generation sequencing. Functionally significant driver mutations were identified and analyzed in 29 of these patients. The most frequently mutated genes included TP53 (72%), MLL3 (28%), and PIK3CA (17%). Using a blood-based tumor mutational burden (bTMB) cutoff threshold of 6.7 mutations per megabase, patients with low bTMB demonstrated a significantly better ORR to anlotinib combined with benmelstobart compared to those with high bTMB (50% *vs.* 7%; *P* = 0.015). Furthermore, low bTMB was associated with a greater PFS benefit (mPFS: 7.3 months *vs.* 4.1 months; *P* = 0.012) (39).

The ETER901 study enrolled a total of 147 patients, who were randomly assigned to either the combination therapy group (benmelstobart plus anlotinib; *n* = 75) or the control group (albumin-bound paclitaxel; *n* = 72). As of December 2024, the primary study results demonstrated a mPFS of 7.85 months in the combination group versus 5.55 months in the control group (HR = 0.70; 95%CI: 0.46-1.06; *P* = 0.1687), representing a 2.3-month prolongation compared to the control. For mOS, the combination group achieved 35.81 months versus 21.03 months in the control group (HR = 0.78; 95% CI: 0.49-1.24; *P* = 0.2625), corresponding to an improvement of nearly 15 months. In terms of safety, the incidence of grade ≥3 drug-related adverse events in the combination group was 56%, primarily involving manageable hypertension and hypertriglyceridemia. These findings were consistent with the known safety profiles of the two drugs, and no new safety signals were identified (40).

Based on these clinical trial results, the benmelstobart and anlotinib combination demonstrates promising efficacy in first-line recurrent or metastatic TNBC.

### 3.7 Esophageal squamous cell carcinoma

ICIs combined with chemotherapy represent the standard-of-care therapy for advanced ESCC; however, considerable unmet clinical needs persist for this patient population. Clinical data indicate that approximately 30% of patients with advanced ESCC fail to respond to first-line immunotherapy. Furthermore, the efficacy of immune checkpoint inhibitor-chemotherapy combinations remains limited (41, 42). Luo et al. conducted a multicenter, multi-cohort, exploratory phase II trial (NCT05013697) to evaluate the efficacy and safety of benmelstobart combined with anlotinib, paclitaxel, and cisplatin as first-line therapy for patients with unresectable locally advanced, recurrent, or metastatic ESCC. Eligible patients received combination therapy with benmelstobart, anlotinib, paclitaxel, and cisplatin for 4 to 6 cycles. Patients who did not experience disease progression subsequently received maintenance therapy with benmelstobart plus anlotinib until disease progression or unacceptable toxicity. From September 2021 to November 2023, 50 patients were enrolled and received study treatment. With median follow-up of 23.7 months as of April 1, 2024, mPFS was 14.9 months (95% CI: 11.4-not estimable [NE]) and the 1-year PFS was 58.5% (95% CI: 41.9%-71.9%). Among 50 patients, confirmed ORR was 72.0% and DCR was 84.0%. Median DoR of 36 responders was 16.2 months (95% CI: 10.2-NE). At the cutoff date, 31 patients remained alive; mOS was not reached (95% CI: 13.2 months-NE) with 1-year OS of 74.8% (95% CI: 59.8%-84.8%). 46 (92.0%) patients reported TRAEs, with 37 (74.0%) were grade ≥3 (1).

The ALTER-E003 trial (NCT05038813) was a multicenter, single-arm phase II study that enrolled 46 patients with advanced, first-line ESCC. Patients received anlotinib (12 mg, orally on days 1-14) combined with benmelstobart (1200mg, intravenously on day 1) of each 21-day cycle until disease progression or unacceptable toxicity. From March to September 2022, 46 patients with advanced ESCC were enrolled in this study. All enrolled patients received at least one cycle of treatment and were included in both the safety and efficacy analyses. As of the data cutoff date (February 29, 2024), the confirmed ORR was 56.5% (95% CI: 41.1-71.1), and the DCR was 91.3% (95% CI: 79.2-97.6). Among the 26 confirmed objective responders, the median DoR was 17.81 months (95% CI: 12.68-20.60), the mPFS was 15.74 months (95% CI: 9.03-21.91), and the mOS was 20.57 months (95% CI: 14.65-not reached [NR]). In terms of safety, the overall incidence of TRAEs was 93.5% (43/46), and the incidence of grade≥3 TRAEs was only 28.3% (13/46). Common grade≥3 TRAEs included hypertension (10.9%), hyponatremia (4.3%), and decreased neutrophil count (4.3%). There was 1 patient each (2.2%) with proteinuria, palmar-plantar erythrodysesthesia syndrome, and upper gastrointestinal bleeding. No treatment-related deaths occurred. Furthermore, genomic profiling identified a novel predictive mutational signature (TP53+/FAT1+/NOTCH3−) associated with enhanced clinical benefit. Patients harboring this signature (*n* = 32) demonstrated significantly higher ORR compared to other patients (*n* = 9) (65.6% *vs* 11.1%, *P*<0.001), a prolonged mPFS of 12.59 months (17.91 months *vs* 5.32 months; HR = 0.27, *P* = 0.005), and a trend toward improved mOS (not reached [NR] *vs* 9.59 months; HR = 0.29, *P* = 0.006) (43).

### 3.8 Soft tissue sarcoma

Therapeutic options are limited for unresectable and metastatic soft tissue sarcomas (STSs), where chemotherapy and radiotherapy remain the cornerstone approaches for the vast majority of patients with advanced STS (44). Although pharmacotherapy modestly prolongs PFS or OS in patients with STS, the ORR remains suboptimal (45). Therefore, developing novel therapeutic approaches remains an unmet medical need in the management of STS, particularly for advanced-stage disease. Liu et al. conducted a single-arm, phase II clinical trial to explore the efficacy and safety of benmelstobart combined with anlotinib in patients with locally advanced or metastatic soft tissue sarcoma (LA/M STS). Between January 2019 and June 2020, 30 patients were enrolled. The ORR was 36.67% and the DCR was 76.67%. The median PFS was 7.85 months [95% CI 2.89–23.06] and the median OS was not reached (95% CI 10.58–NE). Among the patients with ASPS (12/30, 40%), the ORR was 75% and the median PFS was 23.06 months (95% CI, 8.97–NE). The most common treatment related adverse events were hypothyroidism (76.67%), hypertriglyceridemia (63.33%), hypercholesterolemia (60.00%), and elevated blood lactate dehydrogenase (53.33%). The study showed promising activity in patients with ASPS, also indicating the trend of treatment efficacy in other sarcomas. The toxicity was tolerable. More studies with larger sample size and controlled arm were warranted (46).

Alveolar soft part sarcoma (ASPS) is a rare malignant subtype of STS, accounting for 0.5%-1% of all STSs (47). Previous studies indicate that either anti-angiogenic agents or ICIs demonstrate modest activity as monotherapy in ASPS, however, the clinical benefit remains limited (48, 49). A single-arm, phase 2 study evaluated the efficacy of benmelstobart combined with anlotinib in adults with advanced ASPS. The study enrolled 29 patients, with 28 evaluable (one withdrew due to acute pancreatitis). An objective response was achieved in 82.1% of patients, including 4 complete and 19 partial responses. The median time to response was 2.8 months, and the DOR was not reached, with an estimated median PFS of 35.2 months. No deaths occurred during follow-up, and the combination regimen demonstrated favorable tolerability. AEs were predominantly grade 1 or 2. Anlotinib dose reduction was required in three patients due to grade 2–3 proteinuria, while bemuciximab was discontinued in one patient following an immune-related acute pancreatitis event. Grade≥3 AEs occurred in 13 patients (44.83%), primarily comprising hypertriglyceridemia (13.79%), lipase increased (6.90%), amylase increased (3.45%), and hypertension (3.45%). Analysis of the tumor microenvironment in 7 patients revealed significant differences in tertiary lymphoid structure formation between responders (*n* = 3; CR or near-CR) and non-responders (*n* = 4; PD or no response). Responders also demonstrated a higher proportion of CD20^+^ cells (1.33% *vs*. 0.19%) (50). Previous studies indicate that anti-angiogenic TKIs, exemplified by anlotinib monotherapy, achieve a mPFS of approximately 18 months in ASPS. In contrast, the anlotinib plus benmelstobart combination demonstrated a 3-year PFS rate of ~50% in the ASPS cohort, reflecting a significant extension in mPFS alongside improved quality of life.

### 3.9 Advanced biliary tract cancer

Biliary tract cancers (BTCs) are highly aggressive and heterogeneous malignancies originating from the biliary epithelium (51). Recent breakthroughs in first-line therapy for advanced BTC involve ICI-chemotherapy combinations. The landmark TOPAZ-1 and KEYNOTE-966 trials demonstrated that these regimens significantly extend patient survival, though the OS benefit remained modest at <2 months, indicating substantial room for further therapeutic advancement (52, 53). Zhou et al. reported a pooled analysis of two single-center phase Ib trials (TQB2450-Ib-05 and TQB2450-Ib-08) assessing the efficacy and safety of anlotinib plus benmelstobart in previously treated patients with advanced BTC. The analysis included 66 patients with advanced BTC. Primary tumor sites were predominantly intrahepatic bile duct (46.97%), gallbladder (30.30%), and extrahepatic bile duct (22.73%). Patients across both trials received identical treatment: intravenous benmelstobart (1200 mg every 3 weeks) plus oral anlotinib (10mg once daily, escalated to 12mg if tolerated, administered on a 2-weeks-on/1-week-off schedule). The study results demonstrated a complete response in 2 patients and a partial response in 12 patients, yielding an ORR of 21.21% (95% CI: 12.11-33.02%) in the overall cohort. Stable disease was achieved in 34 patients, with 28 maintaining SD for ≥24 weeks. This resulted in a DCR of 72.73% (95% CI: 60.36-82.97%) and a clinical benefit rate of 42.42% (95% CI: 30.34-55.21%). The mPFS was 6.24 months (95% CI: 4.11-8.25), while the median DoR was not reached. Median OS was 15.77 months (95% CI: 10.74-19.71). AEs occurred in 96.97% (64/66) of patients, with TRAEs reported in 89.39% (59/66). Grade ≥3 TRAEs occurred in 25.76% (17/66) of patients. The most frequent grade ≥3 TRAEs were increased aspartate aminotransferase (7.58%; *n* = 5), increased alanine aminotransferase (6.06%; *n* = 4), and hypertension (6.06%; *n* = 4). Additionally, comprehensive biomarker analyses were performed to identify potential predictors of treatment response. High tumor mutational burden (TMB ≥5 mutations/megabase [mut/Mb]) was associated with superior clinical benefit rate (CBR) (70.8% *vs.* 22.2%; *P* = 0.004), longer mOS (14.32 *vs.* 9.64 months; *P* = 0.009), and prolonged mPFS (7.03 *vs.* 4.06 months; *P* = 0.059) compared to low-TMB tumors. Patients with KRAS mutations exhibited reduced CBR (12.5% *vs.* 58.8%; *P* = 0.045), shorter median PFS (2.02 *vs*. 6.80 months; *P* < 0.001), and diminished OS (10.53 *vs.* 13.13 months; *P* = 0.038). Other molecular alterations—including ARID1A, ARID1B, and FGF/FGFR pathway variants—were identified as potential correlates of treatment efficacy or prognosis (54).

An exploratory phase II trial evaluated first-line benmelstobart plus anlotinib combined with albumin-bound paclitaxel and cisplatin in advanced BTC. As of January 2024, best responses included PR in 12 patients, SD in 6, and progressive disease (PD) in 2. Preliminary efficacy demonstrated an ORR of 60.0% (95% CI: 36.1-80.9%) and a DCR of 90.0% (95% CI: 68.3–98.8%). In the gallbladder cancer subgroup (*n* = 8), ORR reached 75%. At data cutoff, 5 patients had discontinued due to disease progression. The interim mPFS was 8.31 months (95% CI: NE); these survival data remain immature. TRAEs of any grade occurred in 100% (20/20) of patients, with grade ≥3 TRAEs reported in 45% (9/20). The most frequent grade ≥3 TRAEs included decreased white blood cell count (30%), pyrexia (5%), decreased platelet count (5%), fatigue (5%), abdominal pain (5%), and oral mucositis (5%) (55). The benmelstobart plus anlotinib combined with albumin-bound paclitaxel and cisplatin regimen demonstrates promising efficacy and manageable safety in first-line BTC. While immune-chemotherapy combinations in the TOPAZ-1 and KEYNOTE-966 trials achieved ORRs below 30%, this quadruplet regimen yielded an ORR of 60% (reaching 75% in the gallbladder cancer subgroup).

### 3.10 Advanced/metastatic gastric or gastroesophageal junction cancer

Multiple clinical trials, including KEYNOTE-859 and ORIENT-16, have demonstrated that PD-1 inhibitors significantly prolong OS and PFS in patients with PD-L1-positive advanced gastric cancer (GC), progressively reshaping treatment paradigms. Despite these significant advances in GC immunotherapy, clinical benefit remains a major challenge for patients with low PD-L1 expression (56–58). A prospective, single-arm, phase II trial exploring the efficacy and safety of anlotinib combined with benmelstobart and the SOX regimen in patients with HER2-negative, PD-L1 CPS <5 advanced G/GEJ adenocarcinoma was reported at the 2025 American Society of Clinical Oncology Gastrointestinal Cancers Symposium (ASCO GI). From June 2023 to September 2024, a total of 28 patients were enrolled, of whom 27 were evaluable for efficacy and safety. Among the efficacy-evaluable patients, PR was observed in 19 patients (70.4%), SD in 6 patients (22.2%), and 2 patients (7.4%) were not evaluable. The ORR was 70.4% (95% CI: 49.8-86.2), and the DCR was 92.6% (95% CI: 75.7-99.1). As of September 17, 2024, treatment was discontinued in 6 of the 27 patients (three due to PD and three who withdrew consent), while 21 patients remained on treatment. The maximum duration of treatment (DoT) was 15.08 months, and mPFS was not reached. Regarding safety, TRAEs of any grade occurred in 93% of patients (25/27). TRAEs occurring in ≥10% of patients included thrombocytopenia (37%), anemia (33%), leukopenia (22%), hand-foot syndrome (19%), fatigue (15%), abnormal liver function (15%), and decreased appetite (11%). Grade ≥3 TRAEs included thrombocytopenia (4%) and anemia (4%). Compared to conventional regimens, this study demonstrated improved ORR and DCR, along with a manageable safety and tolerability profile (59).

Another phase 2 study investigated first-line benmelstobart plus anlotinib and chemotherapy in HER2-negative unresectable locally advanced/metastatic G/GEJ cancer. Twenty-five eligible patients received benmelstobart plus anlotinib and chemotherapy for 6 cycles, followed by benmelstobart and anlotinib maintenance. Of 24 patients with post-treatment imaging, ORR is 75.0% (95% CI: 53.3%-90.2%), and DCR is 100.0%. The mDoR is 10.9 months. By the date cutoff, the median follow-up is 15.8 months. Median PFS and OS among all 25 patients are 10.3 and 18.2 months, respectively. Survival outcomes are not associated with PD-L1 expression. Lymphocytes, T cells, and CD3^+^CD8^+^ T cells are enriched in patients with long-term response (PFS>12 months). Most common grade≥3 TRAE is neutrophil count decreased (12%) (60). This study shows promising efficacy and safety, representing a potential first-line option in patients with HER2-negative advanced G/GEJ cancer, regardless of PD-L1 expression.

### 3.11 Advanced acral melanoma

Acral melanoma is the predominant melanoma subtype in Asian populations (61). Current immunotherapy yields suboptimal responses in patients with AM, with reported ORR ranging from only 14% to 26% and PFS from merely 3.2 to 4.1 months. A standardized treatment approach for advanced AM has not yet been established (62). A phase Ib study (NCT03991975) evaluated the safety and efficacy of benmelstobart and anlotinib in patients with advanced acral melanoma. Patients received benmelstobart (1200mg every 3weeks) and anlotinib (10mg or 12mg once daily, 2-week on/1-week off) in the dose-escalation and dose-expansion phases. The primary endpoints were dose-limiting toxicity (DLT), maximum tolerated dose (MTD) and ORR. Nineteen patients were enrolled between June 2019 and June 2022. The majority of patients (16 of 19 patients) received anlotinib and benmelstobart as first-line treatment. No DLTs were observed, and MTD was not reached. Eighteen (94.7%) out of 19 patients experienced TRAEs, but most were grade 1 or 2. Grade 3 or greater TRAEs occurred in seven patients (36.8%). The ORR was 26.3% (two complete responses and three partial responses). The disease control rate was 73.7%. The median DoR was 30.3months (95% CI: 5.8-NE). The mPFS was 5.5months (95% CI: 2.8-NE), and mOS was 20.3months (95% CI: 14.8-NE) (63). This represents the first study to investigate the safety and efficacy of a multi-targeted TKI combined with an anti-PD-L1 monoclonal antibody in patients with AM. In this study, combination treatment using anlotinib and benmelstobart was found to have a promising anti-tumor efficacy and a favorable safety profile in patients with advanced AM.

### 3.12 Ovarian cancer

The management of platinum-resistant ovarian cancer remains a persistent clinical challenge. Standard non-platinum regimens demonstrate limited efficacy in this patient population. These include chemotherapy (with or without bevacizumab), immunotherapy (with or without chemotherapy), and PARP inhibitors: only ≤15% of patients achieve an objective response, with a median progression−free survival (mPFS) of merely 3–4 months. (64). Therefore, novel therapeutic approaches need to be explored to address this unmet clinical need in platinum-resistant ovarian cancer. The ACTION trial (NCT04236362) is a phase Ib study of anlotinib plus benmelstobart for platinum-resistant or -refractory ovarian cancer. As of 31 May 2021, thirty-four patients were enrolled and received treatment. The ORR is 47.1%, and the disease control rate is 97.1%. The median DoR has not been reached, and 61.3% of patients have a DoR of at least 8 months. The mPFS is 7.8 months, and the mOS has not been reached. The PD-L1-positive group has an ORR of 25.0%, whereas the PD-L1-negative group has an ORR of 92.9%. Treatment-related grade 3 or 4 AEs occur in 70.6% of patients, with the most common being hypertension (29.4%) and palmar-plantar erythrodysesthesia syndrome (29.4%) (65). Anlotinib plus benmelstobart show promising antitumor activity and manageable toxicities in patients with platinum-resistant or -refractory ovarian cancer. Anlotinib plus benmelstobart has promising antitumor activity and manageable toxicities in patients with platinum-resistant or -refractory ovarian cancer. Accordingly, a phase 3 multicenter randomized trial (ClinicalTrials.gov: NCT05145218) to investigate anlotinib plus benmelstobart as treatment for patients with platinum-resistant or -refractory ovarian cancer is ongoing.

### 3.13 Ongoing clinical trials

In addition to the studies mentioned above, several clinical trials are also currently ongoing (**Table 1**). One randomized, double-blind, parallel-controlled, multicenter Phase II trial (NCT06141226) is designed to compare the efficacy and safety of benmelstobart plus anlotinib and chemotherapy versus benmelstobart plus chemotherapy in advanced NSCLC patients who progressed after first-line chemoimmunotherapy. This study also aims to identify biomarkers associated with treatment response, mechanisms of action or resistance, and overall safety profiles. Another phase III clinical trial (NCT06141226) aimed to evaluate the effectiveness and safety of microwave ablation combined with anlotinib and benmelstobart in patients with advanced hepatocellular carcinoma. Patients were randomly assigned at a one-to-one ratio to receive microwave ablation plus anlotinib and benmelstobart or microwave ablation plus benmelstobart. The primary endpoint is objective response rate (ORR), while secondary endpoints include overall survival, progression-free survival, and disease control rate. Furthermore, a single-center phase II clinical trial (NCT06699498) plans to enroll eligible 45 patients with stage III/IVA squamous cell carcinoma of the head and neck who meet the inclusion and exclusion criteria. The trial intends to investigate the safety and efficacy of neoadjuvant therapy with benmelstobart in combination with anlotinib and chemotherapy for locally advanced squamous cell carcinoma of the head and neck.

**Table 1:** Ongoing clinical trials of “benmelstobart + anlotinib” treatment for malignant tumors.

Registration number	Study title	Primary outcome measures	Secondary outcome measures	Phases
NCT06141226	Randomized, Double-blind, Parallel Controlled, Multicenter Phase II Clinical Trial to Evaluate the Safety and Efficacy of TQB2450 Injection Combined With Arotinib Capsules and Chemotherapy in the Treatment of Advanced Non-small Cell Lung Cancer After Immune Resistance	PFS	ORR, DCR, OS, DoR	2
NCT03910127	A Phase Ib, Multicenter, Randomized Study to Evaluate the Pharmacokinetics, Efficacy and Safety of TQB2450 Combined With or Without Anlotinib in Patients With Advanced Non-small Cell Lung Cancer (NSCLC)	PFS	AEs, ORR, DCR, OS, Pharmacokinetics (PK)	1
NCT06851169	A Two-cohort, Exploratory Clinical Study of Perioperative Benmelstobart Alone or Combined with Anlotinib in Patients with Resectable PD-L1≥50% Non-small Cell Lung Cancer	pCR	MPR, ORR, EFS, DFS, OS	2
NCT04964479	A Randomized, Blind, Parallel Controlled, Multicenter Phase III Clinical Trial to Evaluate the Efficacy and Safety of TQB2450 Injection Combined With Anlotinib Hydrochloride Capsules Versus Pembrolizumab Injection as a First-line Treatment in Patients With Advanced NSCLC	PFS	OS, ORR, DCR, DoR, AEs	3
NCT05913089	A Phase II/III Clinical Study on the Efficacy and Safety of TQB2450 Injection Combined With Chemotherapy or Anlotinib Hydrochloride Capsule in the Perioperative Treatment of Resectable Stage II/III Non-Small Cell Lung Cancer	Major pathologic response (MPR)	OS, EFS, DFS, CR	2/3
NCT03983928	A Phase Ib, Open-label, Single Center, Non-randomized Study for Safety and Efficacy of TQB2450 Combined With Anlotinib in Subjects With Advanced Mutation Positive Non-Small Cell Lung Cancer	ORR	PFS, OS, DCR	1
NCT05346952	A Phase 3, Randomized, Open, Parallel Controlled, Multi-center Study of TQB2450 Injection Plus Chemotherapy Followed by TQB2450 Plus Anlotinib Versus Tislelizumab Plus Chemotherapy Followed by Tislelizumab as a First-line Treatment in Patients With Advanced Non-squamous NSCLC	PFS, OS, ORR, DCR, DoR	AEs	3
NCT06931145	A Real-world Study of Benmelstobart, Anlotinib and Chemotherapy Regimen as First-line Treatment for Extensive-stage Small-cell Lung Cancer	PFS	OS, ORR, DoR, DCR	4
NCT06825208	Etoposide Plus Benmelstobart Followed by Maintenance Therapy of Benmelstobart Plus Anlotinib in First-line Treatment of Elderly Patients with Extensive Stage Small Cell Lung Cancer: a Single-arm, Prospective Trial	PFS	AEs, OS	NA
NCT06897579	A Randomized Controlled, Open-label, Multicenter Clinical Trial Evaluating the Efficacy and Safety of Carboplatin/Cisplatin + Etoposide + Bemarituzumab Followed by Bemarituzumab Combined With Anlotinib Versus Carboplatin/Cisplatin + Etoposide + Tislelizumab Followed by Tislelizumab as First-line Treatment for Extensive-stage Small Cell Lung Cancer	PFS	OS, ORR, DCR, AEs,	2
NCT06978153	Perioperative Treatment With Benmelstobart Combined With Chemotherapy With or Without Anlotinib in Resectable Limited-Stage Small Cell Lung Cancer: A Randomized, Two-Cohort, Multicenter, Phase II Clinical Study	EFS	MPR, pCR, ORR, OS	2
NCT05942508	An Open, Single Arm, Single Center Phase Ib Clinical Trial to Evaluate the Efficacy and Safety of TQB2450 Combined With Anlotinib as Maintenance Therapy in Patients With Limited Stage Small Cell Lung Cancer Without Progression After First-line Radiotherapy and Chemotherapy	AEs	ORR, DCR, DoR	1
NCT05145218	A Multicenter, Randomized, Open, Parallel Controlled Phase III Clinical Trial to Evaluate the Efficacy and Safety of TQB2450 Injection Combined With Androtinib Hydrochloride Capsules Versus Paclitaxel as Weekly Treatment in the Treatment of Recurrent Platinum-resistant Ovarian Cancer	PFS, OS	ORR, DCR, DoR, OS, AEs	3
NCT04665609	Safety and Effectiveness of Thermal Ablation Combined With Anlotinib and TQB2450 Solution for Advanced Hepatocellular Carcinoma	ORR	OS, PFS, DCR, AEs	3
NCT06031480	Study of Combination Treatment Using Anlotinib and TQB2450 in Patients With Advanced Hepatocellular Carcinoma Who Failed Prior Immune Checkpoint Inhibitor Therapies: a Single Arm, Multicenter Clinical Trial	ORR	/	2
NCT05311319	Hepatic Artery Infusion Chemotherapy(HAIC) Combined With Anlotinib and TQB2450 as Adjuvant Therapy in HCC Patients at High Risk of Recurrence After Resection	DFS	OS, AEs, Time to recurrence (TTR)	2
NCT05111366	An Open, Single Arm, Multicenter, Exploratory Phase II Clinical Trial of TQB2450 Plus Anlotinib as Adjuvant Therapy in HCC Patients at High Risk of Recurrence After Resection	1-year Recurrence-free survival (RFS) rate	AEs, OS	2
NCT06475287	The Efficacy and Safety of HAIC Combined With TQB2450 and Anlotinib in Second-line Treatment of Advanced Hepatocellular Carcinoma: an Open, Single Arm Exploratory Study	ORR	AEs	2
NCT04888546	A Single-arm, Multicenter Phase Ib Clinical Trial of the Efficacy and Safety of TQB2450 Injection Combined With Anlotinib Hydrochloride Capsule Neoadjuvant in the Treatment of Resectable Hepatocellular Carcinoma With a High Risk of Recurrence or Metastasis	pCR, ORR	PFS, OS, AEs	1/2
NCT03825705	Phase Ib Clinical Study on Safety and Efficacy of TQB2450 Injection Combined With Anlotinib Hydrochloride Capsule in Patients With Advanced Biliary Adenocarcinoma/Hepatocellular Carcinoma	ORR	AEs	1/2
NCT05812430	The Efficacy and Safety of Anlotinib Plus TQB2450 Combined With Nab-paclitaxel and Cisplatin as First-line Treatment for Advanced Biliary Tract Cancer	ORR	PFS, OS, DCR	2
NCT04809142	A Randomized, Open-label, Parallel Controlled, Multi-center Phase III Study of TQB2450 Injection Combined With Anlotinib Hydrochloride Capsule Versus Chemotherapy as Second-line Treatment in Subjects With Advanced Biliary Cancer	OS	PFS, ORR, DCR, DoR	3
NCT03996408	Phase Ib Study to Evaluate the Pharmacokinetics, Safety and Efficacy of TQB2450 Injection (PD-L1 Antibody) Combined With Anlotinib in Subjects With Advanced Cholangiocarcinoma	DLT, Determine the Recommended Phase II Dose (RP2D), ORR	DCR, PFS, OS, AEs	1/2
NCT05013697	A Multicenter Exploratory Study of Paclitaxel+Cisplatin+TQB2450 Injection Combined With or Without Anlotinib in the First-line Treatment of Advanced Esophageal Squamous Cell Carcinoma	PFS	ORR, DCR, DoR, AEs	2
NCT05038813	A Single-arm, Multi-center Exploratory Clinical Study of Anlotinib Combined With TQB2450 (PD-L1 Inhibitor) in the Treatment of Advanced Esophageal Squamous Cell Carcinoma	ORR	AEs, PFS, DCR, DoR, OS	2
NCT05252078	An Open, Single Arm, Multicenter, Exploratory Phase II Clinical Trial of Anlotinib Hydrochloride Capsules Combined With TQB2450 Injection in Esophageal Squamous Cell Carcinoma Patients as Postoperative Adjuvant Therapy	DFS	1/3-year DFS rate, 1/3-year OS rate	2
NCT06939452	A Single-arm, Multicenter Exploratory Clinical Trial of Anlotinib Combined With TQB2450 and the SOX Regimen as First-line Treatment for Advanced Gastric Cancer With Low PD-L1 Expression	ORR	DCR, PFS, DoR, 1-year OS rate	2
NCT04891900	A Single Arm, Multicenter Phase II Clinical Study of TQB2450 (PD-L1 Inhibitor) Plus Anlotinib Combined With Oxaliplatin, Capecitabine in the First-line Treatment of Advanced Gastric Cancer (GC) or Adenocarcinoma of Esophagogastric Junction (AEG)	ORR	DCR, PFS, OS	NA
NCT04611711	An Evaluation of the Effectiveness and Safety of Decitabine Combined With TQB2450 Injection (PD-L1 Monoclonal Antibody) or Decitabine + Anlotinib Combined With TQB2450 Injection in the Treatment of PD-1 Monoclonal Antibody-resistant Digestive System Tumors I/Phase II Clinical Study	ORR	OS	1/2
NCT06662006	Efficacy and Safety of Second-line Therapy by Nal-IRI/5-FU/LV Chemotherapy Combined with PD-L1 Inhibitor and Multi-target Anti-angiogenic Small Molecule ± SBRT in Metastatic Pancreatic Cancer Patients: a Prospective, Multicenter, Single-arm, Multi-cohort Study	ORR	DCR, mPFS, mOS	2
NCT06621095	Anlotinib Plus Benmelstobart and AG Versus AG in First-line Treatment of Advanced Metastatic Pancreatic Cancer: a Prospective, Randomized Controlled Clinical Trial (ALTER-PA-001)	ORR	PFS, DCR, DoR, OS, AEs	2
NCT06469879	A Randomized, Double-blind, Placebo-controlled Phase III Clinical Trial to Evaluate the Efficacy and Safety of TQB2450 Combined With Anlotinib as Maintenance Therapy in Patients With Limited-stage Small Cell Lung Cancer	PFS	PFS, OS, ORR, DoR, DCR	3
NCT04405505	A Randomized, Positive Parallel Controlled, Multicenter Phase III Study of TQB2450 Injection Combined With Anlotinib Hydrochloride Capsule Versus Paclitaxel for Injection (Albumin Bound) in Subjects With Triple Negative Breast Cancer (TNBC)	PFS	ORR, DoR, DCR	3
NCT06874933	A Prospective, Single-Arm Clinical Study on the Treatment of HR+/HER2- Breast Cancer With Neoadjuvant Chemotherapy Combined With Anlotinib and Benmelstobart Monoclonal Antibody	PCR, RCB 0–1 Ratio	EFS	2
NCT06848439	A Phase II Study of Benmelstobart Combined With Anlotinib and Chemotherapy as Neoadjuvant Therapy Followed by Surgery and Postoperative Radiotherapy in Patients With Locally Advanced Oral Cancer	DFS	ORR, MPR, pCR, OS	2
NCT06699498	Phase II Clinical Study of Neoadjuvant Therapy With Benmelstobart Combined With Anlotinib and Chemotherapy for Patients With Locally Advanced Head and Neck Squamous Cell Carcinoma	MPR	ORR, pCR	2
NCT06429397	A Single-arm, Multicenter, Prospective Phase II Clinical Study of Anlotinib Combined With Benmelstobart in the First-line Treatment of Advanced Pheochromocytoma/Paraganglioma	ORR	PFS, OS, DoR	2
NCT03897283	Phase Ib Study of TQB2450 Combined With Anlotinib in Patients With Advanced Solid Tumors	DLT, Maximum tolerated dose, RP2D	ORR, DCR, PFS, OS, AEs	1

## 4 Safety profile of benmelstobart plus anlotinib combination

Safety profiles of benmelstobart plus anlotinib combination in approved indications are shown in **Table 2**. In the ETER701 trial (NCT04234607) involving SCLC, triple therapy led to grade ≥3 TRAEs in 93.1% of patients, with neutropenia (69.9%) and thrombocytopenia (49.6%) being the most frequent (3). Similarly, in a study on endometrial cancer (NCT04574284), cohort 1 (benmelstobart plus anlotinib) reported grade ≥3 TRAEs in 74.77% of cases, commonly including hypertension (60.75%), hypothyroidism (48.60%), weight loss (46.73%), and reduced white blood cell count (47.66%) (24). Another trial in EC (NCT05481645) observed a 81.58% incidence of grade ≥3 TRAEs with the same combination, where decreased white blood cell count (52.63%), thrombocytopenia (28.9%), and anemia (26.3%) were the most prevalent events exceeding 20% frequency (25). Data from a RCC trial (NCT04523272) indicated that hypertension, proteinuria, hypertriglyceridemia, and hand-foot syndrome were the common grade 3 or higher TRAEs in the benmelstobart-anlotinib group. Moreover, rates of grade ≥3 TRAEs (75.0% vs 74.62%), treatment discontinuation due to TRAEs (12.50% vs 6.44%), and fatal TRAEs (4.92% vs 2.27%) were comparable between treatment arms (4). In summary, the benmelstobart-anlotinib combination demonstrates a manageable and consistent toxicity profile across multiple malignancies, largely reflecting expected immune-related adverse events and class effects of anti-angiogenic agents. Current challenges include the absence of predictive biomarkers for severe toxicity and the need for optimized intermittent dosing strategies. These results highlight the necessity for tumor-specific risk-benefit evaluations and standardized toxicity management to optimize the therapeutic index.

**Table 2:** Safety profiles of benmelstobar plus anlotinib combination across approved cancer types.

Cancer types	Study	Intervention/ Treatment	Most common ≥o level adverse events	incidence of Grade ≥3 TRAEs (%)
Endometrial cancer	TQB2450-II-08 (NCT04574284)	Benmelstobart and anlotinib	Weight loss, white blood cell decreased, blood corticotrophin increased and thyroid stimulating hormone increased	74.77%
Endometrial cancer	NCT05481645	Benmelstobart plus carboplatin+paclitaxel and anlotinib	Decreased white blood cell count, thrombocytopenia and anemia	81.58%
Renal cell carcinoma	ETER100 (NCT04523272)	Benmelstobart+anlotinib	Hypertension, proteinuria, hypertriglyceridemia	N/A
Extensive stage small cell lung cancer	ETER701 (NCT04234607)	Benmelstobart plus anlotinib and etoposide-carboplatin	Neutropenia and thrombocytopenia	93.1%

## 5 Comparison of critical clinical trial data from different ICI-TKI regimens

In addition to the combination of benmelstobart and anlotinib, the clinical efficacy of other ICI-TKI regimens has also been repeatedly demonstrated (**Table 3**). In Endometrial cancer, KEYNOTE-775 (NCT03517449) study show a survival benefit in lenvatinib plus pembrolizumab (66). Although cross-trial comparisons are not directly feasible due to the non-head-to-head nature of the studies, the benmelstobart plus anlotinib combination demonstrated superior OS compared with lenvatinib plus pembrolizumab, while ORR and PFS were generally comparable (3). This further confirms that the combination of immunotherapy and anti-angiogenic therapy represents a standard treatment paradigm for pretreated endometrial cancer. In the treatment of metastatic or unresectable RCC, axitinib plus toripalimab has been shown to significantly improve PFS and OS (22). However, benmelstobart plus anlotinib combination demonstrates advantages in terms of the magnitude of PFS prolongation, improvement in ORR, breadth of subgroup coverage, and robustness of long-term benefit. These differences may be attributed to the multi-target mechanism and intermittent dosing regimen of anlotinib, which enhance efficacy while improving tolerability, as well as the activity of benmelstobart in PD-L1-negative tumors, which expands the population likely to benefit (67). In ES-SCLC, the combination of camrelizumab plus apatinib following induction chemotherapy demonstrated significant antitumor activity, with an ORR of 88.9%, a mPFS of 7.3 months, and a mOS of 17.3 months (68). Notably, patients enrolled in this study had received two cycles of etoposide plus carboplatin as induction chemotherapy prior to initiating the targeted therapy-immunotherapy combination, whereas those in the ETER701 study did not receive induction chemotherapy.

**Table 3:** Comparison of critical clinical trial data from different ICI-TKI regimens.

Cancer types	Study	Phase	Population	Intervention/ Treatment	Efficacy outcomes
Endometrial cancer	TQB2450-II-08 (NCT04574284)	2	Recurrent/metastatic advanced endometrial cancer; failed 1–2 prior lines of therapy	Benmelstobart and anlotinib	OS: 21.72 months; PFS: 8.38 months; ORR: 31.76%
	NCT05481645	2	Previously untreated advanced stage III/IV or recurrent endometrial cancer	Benmelstobart plus carboplatin+paclitaxel and anlotinib	ORR: 86.1%; mPFS: Not reached; mOS: Not reached
	KEYNOTE-775 (NCT03517449)	3	Advanced, recurrent, or metastatic endometrial cancer; previously treated	Lenvatinib plus pembrolizumab	OS: 18.7 months; PFS: 7.3 months; ORR: 33.8%
Renal cell carcinoma	ETER100 (NCT04523272)	3	Previously untreated advanced clear-cell RCC; ECOG PS 0 or 1	Benmelstobart +anlotinib	ORR: 72%; PFS: 19.0 months; OS: Not reached; DCR: 95%; mDOR: 17.9 months; 1-year PFS rate: 65%; 2-year PFS rate: 42%; 1-year OS rate: 90%; 2-year OS rate: 72%
	RENOTORCH (NCT04394975)	3	Previously untreated intermediate-/poor-risk advanced clear cell RCC; ECOG PS 0 or 1	Axitinib plus toripalimab	ORR: 56.7%; PFS: 18.0 months; OS: Not reached; 1-year PFS rate: 62.7%; 1-year OS rate: 90.5%
Extensive stage small cell lung cancer	ETER701 (NCT04234607)	3	Treatment-naive ES-SCLC; ECOG PS 0 or 1	Benmelstobart plus anlotinib and etoposide-carboplatin	ORR: 81.3%; PFS: 6.9 months; OS: 19.3 months; DCR: 90.7%; mDOR: 5.8 months; 6-month PFS rate: 59.1%; 1-year PFS rate: 27.9%; 1-year OS rate: 64.1%; 18-month OS rate: 50.7%
	NCT05001412	2	Treatment-naive extensive-stage ES-SCLC; ECOG PS 0 or 1	Camrelizumab, apatinib plus etoposide and carboplatin	ORR: 88.9%; PFS: 7.3 months; OS: 17.3 months; DCR: 97.2%; mDOR: 5.4 months; mTTR: 1.5 months; 1-year OS rate: 63.4%

^*^The above data are not head-to-head study data and are for reference only.

The efficacy of combining anlotinib with ICI therapies has also been repeatedly demonstrated in preclinical animal models and cellular assays (69–72). For example, using lung cancer xenograft models, flow cytometry, and immunofluorescence staining, Luo et al. (69) showed that anlotinib promotes CD8^+^ T cell infiltration through upregulation of chemokine CCL5, thereby potentiating PD-1/PD-L1 blockade therapy. Yuan et al. (70) demonstrated that anlotinib downregulates PD-L1 expression via activation of the cGAS-STING/IFN-β pathway, thereby suppressing the proliferation, migration, and immune evasion of gastric cancer cells in both cell lines and a nude mouse xenograft model.

## 6 Biomarkers for prognosis prediction

The combination of benmelstobart and anlotinib has demonstrated promising efficacy across multiple tumor types. However, the identification of predictive biomarkers for this regimen is still in its early stages. Here, we summarize candidate biomarkers and their potential clinical value, drawing insights from both clinical studies and mechanistic research.

### 6.1 PD-L1 expression level

PD-L1 expression is a well-established biomarker for ICI-based therapies, and its relevance extends to the benmelstobart plus anlotinib combination (73). During treatment, ICIs influence the tumor microenvironment and may indirectly affect PD-L1 expression. In the phase III ETER100 trial (first-line advanced clear-cell RCC), the combination demonstrated significant efficacy across PD-L1 expression subgroups. Notably, patients with PD-L1 combined positive score <1% (PD-L1-negative) still achieved a mPFS of 20.1 months, indicating that PD-L1 negativity does not exclude patients from benefiting from this regimen (4). This finding aligns with the TQB2450-II-08 trial in recurrent/metastatic endometrial cancer, where non-MSI-H/dMMR (predominantly pMMR) patients-who often have low PD-L1 expression-attained an ORR of 31.76% and mOS of 21.72 months with benmelstobart plus anlotinib. Given the heterogeneity of tumors, PD-L1 expression may vary significantly across different regions within the same tumor, and a single biopsy may not fully capture the overall expression profile. Therefore, the role of PD-L1 as a predictive biomarker still requires further validation through larger sample sizes and standardized PD-L1 detection platforms.

### 6.2 Circulating tumor DNA

ctDNA analysis effectively captures genomic mutation burden and intratumoral heterogeneity, and enables longitudinal monitoring to reflect dynamic changes in tumors. Studies have shown that ctDNA profiling provides genomic insights into the underlying causes of differential treatment responses, with low ctDNA fraction and a simple tumor mutational profile being associated with better treatment response and prognosis (74). Based on the ALTER0303 study, a tumor mutation index (TMI) was developed as a predictive model. The study found that patients with a low TMI and without IDH1 exon 4 mutations derived greater survival benefit from anlotinib treatment. While significant progress has been made in ctDNA technology, several key areas, including limitations in sensitivity and biological variability in tumor shedding, still require further refinement before its full potential in early-phase clinical studies can be realized.

### 6.3 Mismatch repair status

MMR status is a key biomarker in endometrial cancer, and several clinical trials have explored how it influences response to benmelstobart plus anlotinib (3, 15, 17, 18). In the phase II TQB2450-II-08 trial, patients were stratified by MMR status: those with MSI-H/dMMR tumors received benmelstobart alone, while those with non-MSI-H/dMMR (pMMR) tumors were given benmelstobart plus anlotinib. The combination showed meaningful activity in pMMR patients (ORR 31.76%, PFS 8.38 months), addressing an important unmet need in this subgroup that has historically responded poorly to immunotherapy (17). In contrast, the phase II trial of benmelstobart plus carboplatin/paclitaxel ± anlotinib in first-line advanced/recurrent endometrial cancer showed that pMMR patients derived significant PFS benefit from the addition of anlotinib (HR = 0.35, 95% CI 0.15-0.79), further supporting that the combination can overcome the immunotherapy resistance associated with pMMR status. For MSI-H/dMMR patients, benmelstobart monotherapy already showed promising results in the TQB2450-II-08 trial (ORR 34.38%, OS 27.66 months) (18). Whether adding anlotinib provides additional benefit in this subgroup remains an open question for future studies.

### 6.4 Composition of immune cells in tumor microenvironment

The composition and dynamic changes of TME are closely related to tumor efficacy and prognosis (75). Preclinical and clinical evidence suggests that anlotinib’s multi-target anti-angiogenic activity (targeting VEGFR, PDGFR, FGFR) remodels the TME to enhance ICI efficacy, and TME-related biomarkers may predict this synergy. High-density infiltration of CD8^+^ T cells typically indicates a “hot tumor” phenotype and is associated with enhanced sensitivity to immunotherapy and favorable prognosis (76). In contrast, M2-type macrophages (e.g., CD163^+^) are often linked to immunosuppression and poor prognosis. Cancer-associated fibroblasts contribute to the immunosuppressive tumor microenvironment by secreting immunosuppressive factors (77). In the phase III ETER701 trial, benmelstobart plus anlotinib and chemotherapy improved outcomes across subgroups with different metastatic burdens (e.g., liver metastasis, bone metastasis), which may reflect TME normalization induced by anlotinib (2). Furthermore, in the TQB2450-II-08 trial, the combination’s efficacy in endometrial cancer patients with prior pelvic radiation (a factor altering TME) suggests resilience to TME-related immunosuppression, warranting further investigation of TME-derived biomarkers (e.g., CXCL9, CCL5) in future correlative studies.

## 7 Limitations of current benmelstobart plus anlotinib combination, challenges and future perspectives

Despite promising efficacy across multiple malignant tumors, the benmelstobart plus anlotinib combination faces inherent limitations and clinical challenges that need addressing to optimize its therapeutic potential. Meanwhile, advances in tumor biology and therapeutic innovation offer clear directions for future development, enabling more precise and effective treatment strategies.

Current evidence for the combination largely comes from single-arm trials or subgroup analyses of randomized studies, lacking large-scale head-to-head comparisons with other standard immune-targeted regimens (29, 34, 50). This limits definitive conclusions on its comparative effectiveness and treatment algorithm positioning, especially in tumors with multiple available immune-targeted options. Efficacy predictability remains suboptimal, as many patients develop primary or acquired resistance while underlying mechanisms such as alternative angiogenic pathway activation and immune microenvironment exhaustion are not fully understood (78–80). Safety concerns persist, with grade ≥3 treatment-related adverse events including hypertension, hematological toxicities and immune-related pneumonitis leading to dose reductions or discontinuation in some cases. No reliable biomarkers for severe toxicities exist to guide proactive risk management. Long-term response durability is another issue, with progression common during maintenance phases. Finally, cost-effectiveness and accessibility barriers hinder widespread use, particularly in resource-limited settings.

To address these limitations, future research should focus on three core areas: biomarker-guided precision therapy, optimized dosing strategies and innovative combination regimens, all aimed at enhancing efficacy, improving safety and expanding applicable populations.

Robust predictive biomarkers are critical for refining patient selection. Currently, PD-L1 expression, MMR status and tumor mutational burden show preliminary efficacy correlations, but their utility is limited by detection heterogeneity and lack of prospective validation. Large-scale, multi-center studies should develop integrated biomarker panels incorporating genomic, transcriptomic and tumor microenvironment markers to accurately identify responders. Validating subtype-specific markers will enable tailored treatment and reduce unnecessary toxicities.

Optimizing dosing schedules is key to balancing efficacy and safety. Current regimens use fixed administration patterns, but individual differences in metabolism and tolerance demand personalized adjustments. Intermittent anlotinib dosing may reduce cumulative toxicity while preserving anti-angiogenic and immune-modulatory effects, supported by preclinical data showing enhanced T cell infiltration (10). Ongoing trials explore this approach in specific populations. Therapeutic drug monitoring to adjust doses based on plasma concentrations could improve tolerability, especially in patients with comorbidities. Dose-escalation studies may also identify optimal regimens for distinct patient groups.

Innovative combinations with other modalities can overcome resistance and boost efficacy. Combining with radiotherapy synergizes via immunogenic cell death and vascular normalization, with clinical trials exploring neoadjuvant use in certain tumors (72). Integrating CAR T cell therapy addresses poor infiltration in solid tumors, warranting trials in highly angiogenic tumors. Targeting additional immune checkpoints or immunomodulatory molecules can reverse tumor microenvironment immunosuppression in primary PD-(L)1 resistance cases (81). Combining with metabolic regulators to target tumor microenvironment acidosis or cholesterol metabolism may further enhance anti-tumor immunity, a strategy applicable across multiple tumor types given the conserved role of metabolic reprogramming in cancer (82).

In conclusion, the combination of benmelstobart and anlotinib represents a promising immuno-targeted strategy. However, its clinical utility is currently constrained by a lack of robust comparative data, unpredictable treatment responses, safety concerns, and limited accessibility. Advancing translational and clinical research in key areas will be essential to overcoming these challenges. These areas include the development of precision biomarkers, optimization of dosing strategies, and exploration of novel combination regimens. With continued progress, this regimen has the potential to become a cornerstone of personalized therapy for a broad spectrum of malignancies.
